# The impact of salinity on mycorrhizal colonization of a rare legume, *Galactia smallii,* in South Florida pine rocklands

**DOI:** 10.1186/s13104-017-3105-8

**Published:** 2018-01-02

**Authors:** Klara Scharnagl, Vanessa Sanchez, Eric von Wettberg

**Affiliations:** 1 Agroecology Program, Department of Earth and Environment, Florida International University, 11200 SW 8th St, Miami, FL 33199 USA; 2 Plant Biology, Michigan State University, 612 Wilson Rd, East Lansing, MI 48824 USA; 3 Biological Sciences, Florida International University, Miami, FL 33199 USA; 4 Plant and Soil Science, University of Vermont, Burlington, VT 05403 USA

**Keywords:** *Galactia smallii*, Pine rockland, Soil mutualists, AMF (arbuscular mycorrhizal fungi), Saline soils, Sea level rise

## Abstract

**Objectives:**

The success of restoration plantings depends on the capacity of transplanted individuals or seeds to establish and reproduce. It is increasingly recognized that restoration success depends quite heavily upon biotic interactions and belowground processes. Under stressful abiotic conditions, such as soils salinized by storm surge and sea level rise, symbiotic interactions with soil microbes such as mycorrhizae may be critically important. In this study, we investigate the impact of salinity on percent colonization of roots by arbuscular mycorrhizal fungi, in addition to the impacts of this colonization on plant fitness under saline conditions. Fifty *Galactia smallii* plants from an ex situ collection were subjected to a salinity treatment for 6 weeks, and 50 plants were untreated. Plants were harvested and assessed for percent colonization by arbuscular mycorrhizal fungi, nodule number, shoot and root dry biomass, and micronutrient content.

**Results:**

Colonization by arbuscular mycorrhizae was higher in plants in the salinity treatment than in untreated plants; plants in the salinity treatment were also found to have a lower root:shoot ratio, and higher phosphorus and nitrogen levels. These results support the importance of arbuscular mycorrhizal fungi in restoration efforts of endangered plants in fragmented and threatened ecosystems, such as pine rocklands.

## Introduction

Pine rocklands are a dry forest ecosystem unique to South Florida, Cuba, and the Bahamas. In South Florida, pine rocklands are dominated by the trees *Pinus elliotti* and *Serenoa repens*, yet are host to a large diversity of smaller understory plants, many of which are endemic to this ecosystem [1]. Pine rocklands once covered a large expanse of the southeastern Florida peninsula, yet today stand at two percent of their original extent and are highly fragmented [2]. Many fragments are very small (under a hectare), and only some of the fragments are protected [3].

Legumes with the capacity to symbiotically obtain nitrogen and phosphorus can be critical to low fertility pine rockland systems. One endangered legume is *Galactia smallii*, or Small’s milkpea, a procumbent understory legume endemic to the pine rockland ecosystem [4]. *Galactia smallii* is critically endangered as a consequence of habitat fragmentation [5, 6]. Although endangered, very little is known about this species. Fairchild Tropical Botanic Garden has established a program to replant *Galactia smallii* in pine rocklands. One potential factor in restoration is the belowground community [7]. Arbuscular mycorrhizal fungi are known to help plants deal with a variety of environmental stressors [8, 9]. In pine rocklands, where available phosphorus (Pi) readily binds to the calcium carbonate of the limestone, the ability to acquire phosphorus is critical [10].

We were interested in the role that AMF play in the recovery of rare native legumes in South Florida [11]. Furthermore, we were interested in whether symbionts such as AMF help their host plants combat salinity stress [12]. A common threat to pine rocklands in South Florida is sea level rise due to global climate change [13]. It is predicted that sea level may rise as much as a meter in the coming century [14]. This would engulf the many parts of South Florida that are only a meter above mean sea level and increase salinization of soils throughout the peninsula [14, 15]. A shadehouse study was conducted to investigate the effects of the addition of a saline solution on the AMF relationship of the *G. smallii* plants.

## Main text

### Materials and methods

In 2009, nearly 100,000 *Galactia smallii* individuals were observed near the Homestead Airforce Base [6, 16]. Since development is planned for the base, conservation efforts are underway to relocate some of these individuals. As a part of this effort, in January 2012, 500 adult plants from the Homestead Airforce Base were collected and moved into ex situ conservation at Fairchild Garden. These plants are being maintained in an open-air rare plant nursery for seed collection. During the 2012 collection effort, plants were dug up with as much of their original soil as possible, which was then mixed with standard potting soil (with sterilized composted bark, coconut coir and perlite) and placed in pots (1 L volume). The root systems had 3 months to adjust to potting before the experiment began. One hundred plants from this collection were used.

These 100 plants were placed in a shade house on raised benches, receiving periodic watering from sprinklers connected to a groundwater source. We selected this sample size to retain the other translocated plants to establish a conservation nursery. For 6 weeks, these 100 plants received an addition (2 mL per plant) of P/N-limited Hoagland’s solution [17] each week. Fifty of the plants received a salt treatment: 4–5 mL of 100 ppm saline solution using stock sodium chloride and double deionized water. This salinity treatment mimics the seasonal rise in salinity that can occur in Florida coastal pine rockland patches due to salt water intrusion into the aquifer at the end of the dry season (April–June), or the rise in salinity that can occur from storm-associated salt water surges [5, 15]. Our salt dosage was intended to be sub-lethal, as would be typical of an upland habitat in southern Florida that often occurs on higher ground that is over two meters above sea level. The other 50 plants did not receive a salt treatment. Treatments were made once per week for 6 weeks. The saline treatment would wash away when the plants were watered, acting more like a pulse of saline stress rather than a constant saline stress.

After 6 weeks the plants were harvested. Their roots were washed with tap water, soil samples were collected from each pot, and roots and shoots were separated for weighing and identification of associated soil microbial symbionts. Once root tips collected for analysis, both roots and shoots were dried overnight in an 80 °C drying oven, and weighed.

Twenty root tips, 1–3 cm in length, were randomly collected from each individual *G. smallii* plant’s roots and placed in 10% KOH and left overnight. Root tips were washed three times with tap water, bleached in chlorine for 1–2 min, and washed three times with tap water. Root tips were acidified with 1% HCl, and placed in 0.05% Trypan blue solution overnight. Finally, root tips were removed from Trypan blue, rinsed with tap water, and observed under a compound microscope [18].

Percent of colonized roots for each plant was calculated as:

$$\% {\text{ of colonized roots}} = [\# \,\, {\text{of roots observed with AMF/total }} \# {\text{ of roots observed}}] \times 100.$$

Root colonization includes the observation of arbuscules, vesicles, hyphae or hyphal coils within root cells. Spores and propagules observed around the root tips were not considered observable evidence of colonization. We also counted nodules to assess the association with rhizobial bacteria.

Soil samples were weighed, rinsed with tap water and sieved through 1 mm, 250 µm and 35 µm sieves in order to capture spores. 0.5 mL of spore solution was transferred to filter paper on a slide, and observed under 100× magnification. Spores were counted, and identified to genus level using morphological characteristics [19, 20]. In order to determine number of spores per gram soil, simple stoichiometry was used to balance the number of spores per 0.5 mL solution to the total volume of spore solution per sample (50 mL) and the initial weight of the soil sample (ranging between 12 and 24 g wet weight). We compared percent of roots that were colonized, spore count per gram of soil, number of fungal genera and diversity index between treatments with a two-sample t-test in PROC GLM in SAS 9.3 [21].

Plant dry biomass was used as a proxy for plant fitness. Shoots and roots were dried and weighed separately. We used an ANOVA framework in PROC GLM to test for differences between AMF colonization and salinity treatment in total plant biomass and root:shoot ratio.

Leaf samples were collected from each plant, dried, and homogenized. Homogenized leaf tissue was then digested in 70% nitric acid, heated at 90 °C, digested in hydrogen peroxide, and diluted in deionized water to 10 mL. One milliliter of this solution was re-diluted in 5% nitric acid solution and analyzed on an Inductively Coupled Plasma Optical Emission Spectrometer to assess micronutrient levels. Some homogenized leaf tissue was separated and analyzed on a C/N Analyzer (LECO) for percent carbon and nitrogen. Comparisons were made between the saline and non-saline treatments, with % colonization and number of nodules as covariates, in a ANCOVA in PROC GLM. Because of the small number of comparisons, we did not correct for multiple tests.

## Results

**Fig. 1:**
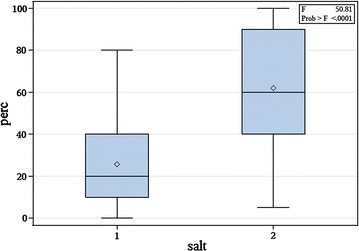
Percent colonization of *Galactia smallii* roots by arbuscular mycorrhizal fungi (*perc* percent colonization;* salt 1* no salt treatment; *salt 2* salt treatment, 100 mM NaCl)

All plants had AMF association (N = 100). Each plant had a percent colonization value between 10 and 100% (Fig. 1), nodule count ranged from 0 to 69 nodules per plant, spore count per gram soil ranged from 30 spores to 223 spores, and the number of genera represented in each sample ranged from 1 to 4, with a total of 6 genera represented overall. Mycorrhizal structures varied extensively, including any combination or single occurrence of the following: large vesicles, small vesicles, arbuscules, extensive hyphae, hyphal coils, and narrow hyphal strands. However, we did not observe any variation in the presence of these structures between salt treated and non-salt treated plants (P > 0.05).

Based upon morphological observations of spores at 100× magnification, six genera within Glomeromycota were represented; *Acaulospora, Gigaspora, Glomus, Paraglomus, Entrophospora,* and *Scutellospora*. We were not able to identify fungi based on spore characters more finely than the level of genus.

We observed higher percent colonization of roots in the salt treatment group than in the untreated group (62% vs 33%, t = 36.18, P < 0.0001). Average nodule number was 14.26 in the salt treatment and 17.18 in the untreated group (not significant). The presence and number of nodules had a significant impact on the number of mycorrhizal spores per gram soil (P < 0.0083), nitrogen content in the leaves (P < 0.0015) and the C:N ratio in the leaves (P < 0.0063) across treatments. We did not observe differences in spore count per gram (96 vs 103, t = 1.28, P = 0.44), number of genera present (2.22 vs 2.24 t = 0.16, P = 0.90) or diversity index (0.369 vs 0.373, t = 0.18, P = 0.88) between treatments.

**Fig. 2:**
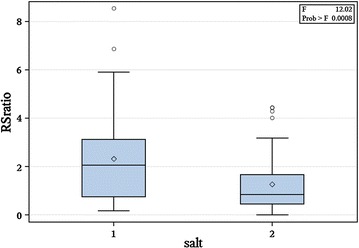
Root to shoot ratio of *Galactia smallii* in no salt treatment [salt = 1] and salt treatment (100 mM NaCl) [salt = 2]

For most of the micronutrients measured, the means do not vary significantly between treatments (Tables 1, 2). However, there were significant differences in phosphorus and sodium across salt treatments (Table 2). We used plant dry biomass as a proxy for plant fitness (Fig. 2). Total plant dry weight (g) was significantly affected by percent colonization across treatments (P < 0.0001). Root:shoot ratio varied among treatments (P < 0.027), with higher root:shoot ratio in the no salt treatment (average 2.32), and lower in the salt treatment (average 1.27).

**Table 1:** Means and standard deviations of 13 key elements in *Galactia* leaf tissue across saline and non-saline growth treatments

	Al	B	Ca	Cu	Fe	K	Mg	Mn	Mo	Na	Ni	P	Zn
Mean, no salt	0.044	0.016	3.55	0.05	0.016	1.48	0.458	0.005	0.0006	2.05	0.004	0.28	0.01
SD	0.024	0.003	1.52	0.015	0.006	0.78	0.15	0.008	0.0003	0.28	0.002	0.34	0.009
Mean, salt	0.061	0.012	3.24	0.018	0.017	1.17	0.53	0.005	0.0002	1.88	0.005	0.72	0.01
SD	0.06	0.006	1.12	0.002	0.009	0.69	0.19	0.007	0.0002	0.285	0.001	0.593	0.009

**Table 2:** Analysis of variance in 13 key elements across saline and non-saline treatments

	Al	B	Ca	Cu	Fe	K	Mg	Mn	Mo	Na	Ni	P	Zn
Salt	0.04	0.42	0.01	3.27	2.05	0	1.36	0.16	2.56	10.28**	0.01	10.67**	0.29
Col	0.61	2.29	17.03*	0.27	11.7**	0.07	3.67	10.17**	6.79*	1.74	1.67	0.98	0
Nodu * salt	0.61	1.59	11.15*	0.69	5.97*	0.2	6.34*	5.04*	0.39	3.46	0.62	3.43	0.63
Col * salt	0.13	0.04	5.86*	0.2	2.07	0.02	0.87	3.68	3.63	1.08	0.5	0.89	0.1
Col * nodu	1.07	4.98*	16.14*	0.14	5.87*	0.09	5.9*	4.86*	0.74	4.18	2.19	0.75	0.18
Col * nodu * salt	1.03	0.54	4.1	0.15	1.26	0.17	1.5	1.38	0	0.06	0.23	0.58	0.75

*Col* percent colonization, *Nodu* nodulation, and interactive effects were considered as co-variates

F values are reported, with * < 0.05, ** < 0.01, *** < 0.001, **** < 0.0001 indicating levels of significance. The numerator and denominator degrees of freedom are 1,12 respectively

## Discussion

The individuals of *Galactia smallii* under salt treatment had much higher overall root colonization by AMF than those individuals grown under normal conditions. However, all individuals had at least 10% colonization by AMF. This implies that under normal conditions, *G. smallii* associate with arbuscular mycorrhizal fungi. Soil fungi in general have a higher tolerance to salinity than plants [22]. When a stressor such as salt is imposed on the *G. smallii* plants, there is a strong increase in AMF association with microbes already present in the soil and a decrease in investment in root biomass. This may be a trade-off employed by the *G. smallii* plants under stressful conditions, as a way to conserve belowground carbon allocation. As the plants invest more in AMF associations and less in root biomass, the AMF replace the role of the roots in the soil, taking up essential water and nutrients for the plants.

Our salt treatment mimicked exposure to salinity in pine rockland habitats in South Florida. Salinity exposure in these upland habitats is often brief, occurring either at the end of the dry season (May–June) when the freshwater lens that rests above the underlying saline groundwater in south Florida is depleted, or following storm surge from a hurricane (e.g., [15]). We observed no plant mortality during this experiment, and the salt treatment did not cause leaf yellowing or other signs of acute salt stress [23]. However, as more frequent and intense storms increase [24], gaining a more thorough understanding of associations in the rhizosphere will become increasingly critical for conservation.

## Limitations

The primary limitations of this study are the use of potted plants that originated from a pine rockland. As an endangered species that is difficult to germinate this was our best option to get material. However, it makes the initial size a co-variate for which it is very difficult to control and means that the colonization of roots may have occurred before we imposed treatments. Furthermore, our treatments occurred over a short time period. This can mimic storm surge, which is a major threat to these systems, but it may affect the extent to which roots could turn over fungal partners from before the start of the study. Another limitation is that we only performed morphological identification of fungi.

## Abbreviations

AMF: arbuscular mycorrhizae
Pi: phosphorus
KOH: potassium hydroxide
PROC GLM: general linear model
ANOVA: analysis of variance
INVAM: International Culture Collection of Arbuscular Mycorrhizal Fungal
LECO: Laboratory Equipment Company
